# “It Hits the Spot”: The Impact of a Professional Development Program on English Teacher Wellbeing in Underdeveloped Regions

**DOI:** 10.3389/fpsyg.2022.848322

**Published:** 2022-06-20

**Authors:** Xiaojing Wang, Zehang Chen

**Affiliations:** School of Foreign Languages and Literature, Beijing Normal University, Beijing, China

**Keywords:** professional wellbeing, primary school teachers in underdeveloped areas, professional development program, English teachers, rural school

## Abstract

In poor and remote areas, teachers frequently encounter serious ongoing challenges and experience teacher exhaustion due to the uneven distribution of resource supply, and a shortage of professional support. Maintaining teachers’ professional wellbeing in an unfavorable teaching environment has become a major challenge. The current study explores teachers’ professional wellbeing in the context of a five-month-long professional development program designed for teachers in areas of deep poverty in China. This study adopted the method of narrative enquiry and collected diaries written before and after the program from 46 primary school English teachers. Based on the manually analyzed data, it was found that teachers’ overall wellbeing at work was enhanced in terms of their professional meaning, engagement, and achievement. The systematic analysis of the diaries helped to better understand how the program exerted such an effect on teachers’ learning experiences and their wellbeing.

## Introduction

Due to rapid sociocultural changes taking place in regard to educational practices, a growing body of research has contributed to investigating how teachers’ sense of wellbeing affects their working commitment in various contexts (Gibbs and Miller, 2014; Day and Hong, 2016; Tang et al., 2018). There is no denying that positive wellbeing can help retain teachers who are working in high-poverty/low-income areas, since these teachers require more emotional engagement in response to the ever-changing indigenous demands (Day and Hong, 2016). In poor and remote regions, teachers frequently encounter serious ongoing challenges and experience teacher exhaustion due to the uneven distribution of resource supply, and a shortage of professional support (Gao and Xu, 2014; Liu et al., 2021).

Teachers in underdeveloped rural areas of China are part of the group who experience the abovementioned challenges, particularly English language teachers, as they are easily marginalized and gradually lose their intrinsic connections with the local rural community (Eckert and Petrone, 2013), which leads to increased teacher turnover. As a result, some remote schools in China encounter a serious shortage of qualified English teachers. The extensive related literature identifies various reasons for poor teacher retention and mostly points to a link to negative teacher wellbeing (e.g., Yan and He, 2011; Yiu and Adams, 2013). It is acknowledged that as long as teachers find their teaching rewarding, any “negative emotions seem marginal” (Zhang, 2021, p. 205). Therefore, improving teacher wellbeing is a high priority in underdeveloped regions. One of the effective approaches to improving teacher wellbeing is to offer them teacher training opportunities. Tang et al. (2018) show that teachers with a greater number of professional development opportunities are certainly content and more likely to provide high-quality teaching as they perceive themselves as having a higher level of wellbeing at work. Teachers in poor and remote regions have many opportunities to attend professional development programs (PDPs) run by local educational authorities or teacher training universities to enhance their professional knowledge and pedagogical skills and alleviate burnout at work (Reagan et al., 2019; Li et al., 2020). Unfortunately, there is little research that explores teachers’ authentic experiences and emotions while attending these training programs and how their professional wellbeing is affected and changed through taking such programs.

Due to the above facts, the purpose of the current study is to explore the impact of PDPs on rural primary school English teachers’ professional wellbeing and to identify the driving factors of change, if any exist. The findings could help identify effective interventions in teacher education in rural contexts and contribute to the theoretical knowledge on how such interventions could help teachers regarding the disadvantaged working environment.

## Literature Review

### Teachers’ Professional Wellbeing

Wellbeing is closely related to an overarching concept of healthy living (Chang et al., 2017). Most definitions of wellbeing show agreement that the term comprises physical, mental, emotional, intellectual and spiritual aspects that extend across stages of a lifetime, and the term is also context-specific (Chang et al., 2017; O’Brien and Guiney, 2021). Seligman (2011, 2018) proposed a five-pillar model to illustrate the construct of wellbeing, including positive emotions, engagement, relationships, meaning, and achievement (abbreviated as PERMA). Positive emotions are a prime indicator of flourishing, which can be learned to promote wellbeing. Engagement involves the pursuit of happiness and the application of strengths. The term relationships encompasses all the various social connections individuals have with others and their community at large. Meaning reflects the personal values that energize people in life, and achievement indicates the ability to strive for goals and manage failure (Falecki et al., 2018; Seligman, 2018). This model comprises both hedonic and eudaimonic wellbeing perspectives.

Teachers’ professional wellbeing includes both affective wellbeing of job satisfaction and other outcome measures of motivation and competence. Its focus is on teacher self-efficacy and job satisfaction, which drives the perception of teachers’ working capability required for everyday practice (Yin et al., 2016; Krekel et al., 2018). This indicates that studying teachers’ professional wellbeing emphasizes the lasting and sustainable eudaimonic perspective; thus, positive emotions in the PERMA model, encompassing “the hedonic feelings of happiness such as joy and contentment,” should be excluded (Morgan and Simmons, 2021, p. 3). Additionally, in the teaching context, forming relationships with students, parents, colleagues is one of the core aspects of evaluating teachers’ engagement at work. Therefore, the current study adopts a three-pillar model to define teachers’ professional wellbeing, including meaning derived from teaching, engagement in work-related activities (including forming relationships at work), and achievements as a teacher. In accordance with Morgan and Simmons’ (2021) claim, the current model could be considered relevant to teachers’ professional wellbeing in any teaching workplace. Using this model, it is more likely to identify how specific social and cultural values affect teachers’ perceptions and evaluations of their career lives, which in turn affects their professional wellbeing (Gibbs and Miller, 2014).

### Rural Teachers’ Professional Wellbeing

The stressful nature of teaching may contribute to low levels of professional wellbeing and a high attrition rate. Teachers in underdeveloped regions are assumed to have more stressors than those who teach in other geographical regions (Mercer, 2020; Cece et al., 2022).

According to Li et al. (2020), the total number of full-time teachers in rural areas in China is decreasing at a rate of 2.754%. To a certain extent, this could largely account for the decreased levels of professional wellbeing, since teachers with lower levels of professional wellbeing are unlikely to stay in disadvantaged settings (Collie et al., 2016; Chang et al., 2017; Smetackova et al., 2019). Extensive research has been conducted on the sources of the low-level wellbeing of teachers working in economically depressed and geographically remote areas; insufficient resources and amenities, unsatisfactory working environments, reluctant student engagement and the lack of career prospects are considered the major factors (Tang et al., 2018; Jurkowski, 2020; Mercer, 2020). Teachers in these areas have to face rather pronounced challenges, as they need to meet the academic, emotional and social needs of underresourced schools and communities. Thus, many of them harbor negative perceptions at work, and their level of wellbeing is relatively low (Corbett et al., 2021). As a result, they might experience ineffectiveness in teaching engagement and unpredictable mental and physical decline; even worse, they might ultimately leave the current profession. Compared with teachers of other subjects, the foreign language teacher attrition rate in these areas is the highest (Acheson et al., 2016; Mercer, 2020; Corbett et al., 2021).

Sargent and Hannum (2009) suggested that teachers could be more satisfied if they were provided opportunities for personal and professional advancement. This idea has also been supported by Acheson et al. (2016), who discovered that rural teachers find their work not only burdensome but also rewarding when it can help accomplish their career goals. In this case, ongoing professional training programs are in high demand for teachers in underdeveloped regions.

### Professional Development Programs and Teachers’ Professional Wellbeing

Professional development programs, are one of the key strategies for teacher improvement and reshaping their work practices, as they provide teachers with the knowledge to critically and regularly re-examine their teaching performance (Tabatabaee-Yazdi et al., 2018; Cirocki and Farrell, 2019). Well-designed PDPs offer professional resources, bring about positive changes in practicing teachers’ competence and provide important tactics for improving learning (Borg, 2018; Corbett et al., 2021). Previous literature has reported that PDPs that promote changes in professional behaviors are helpful in enhancing occupational wellbeing (Gregersen et al., 2020; Mercer and Gregersen, 2020; Corbett et al., 2021).

A strong emphasis was placed by Cirocki and Farrell (2019) on the vital importance of systematic evaluation of PDPs to better understand “their effect in the form of improved pedagogical practices or more successful learning experiences” (p. 2) and their influence on teachers’ professional wellbeing. Unfortunately, drawbacks have emerged that may hinder the effects of PDPs on teacher wellbeing, including the low level of relevance of the theoretical approaches, the passive role of participant teachers, the lack of official support and the lack of teacher learning communities to assure rural teachers a high-quality experience (Yan and He, 2011; Mercer, 2020). Above all, a variety of PDPs have been designed for the ideal implementation of theories; however, their overly academic nature cannot fully fit into local cultures and learning contexts (Mafora, 2013; Li et al., 2020), which means that they fail to cater to the needs of rural teachers and students (Gao and Xu, 2014; Li et al., 2020). In addition, PDPs are usually delivered in the form of lectures, which means that participant teachers are passively involved and lack appropriate ways to internalize their newly acquired knowledge (Zhang and Wei, 2016). Another key factor addressed by Mercer (2020) is “whether teachers are given time off to attend [PDPs]” (p. 10). The sheer amount of time spent in teaching practices and the relentless pace of work results in notable difficulties in rural teachers’ professional development. Without appropriate official support, PDPs can hardly take effect. Another recurring issue for rural teachers taking PDPs is the lack of an active learning community for professional development. Learning communities could create a social and constructive environment in which to disseminate ideas and stimulate discussions. However, several PDPs fail to establish such learning communities to provide continuous idea exchange for rural teachers (Sargent and Hannum, 2009; Tang et al., 2018; Reagan et al., 2019; Liu et al., 2021).

Despite the recent progress that has been made in regard to investigating rural teacher wellbeing (e.g., Tang, 2018) and studying the impacts of PDPs on teachers’ professional success (e.g., Tabatabaee-Yazdi et al., 2018), some literature gaps still exist in examining the connection between the PDPs employed in high-poverty regions and changes in teachers’ sense of wellbeing. As Corbett et al. (2021) claimed, most of the PDPs’ effects on improving teachers’ professional wellbeing remain unknown or uncertain. Therefore, it is recommended that PDPs be fully understood before they are implemented in order to promote teacher wellbeing, particularly in impoverished settings. To this end, the current study intends to bridge this gap by exploring the changes in wellbeing in a group of rural primary school English teachers during an online PDP and identifying what leads to such changes.

## Methodology

### Research Questions

In terms of the literature reviewed, the research questions for the current study are proposed as follows:

RQ1: Did the professional wellbeing of rural primary school English teachers in this PDP change during the training? If yes, how did it change?
RQ2: What features of the PDP may have affected the change in teachers’ professional wellbeing?

### Research Context

The current study was conducted among teachers from schools located in remote counties in four provinces in Southwest China that are recognized as deep poverty areas^1^ with an unfavorable distribution of resources due to their economic and social underdevelopment. Teachers in these remote areas occasionally lack either content knowledge, as the subjects they are teaching now may not have been their majors in college (Sun and Ma, 2015), or pedagogical content knowledge, as their teaching skills may not suit the present needs of the students (Tabatabaee-Yazdi et al., 2018). Accordingly, since 2016, *The Thirteenth Five-Year Plan for Educational Poverty Alleviation* has been promulgated in these areas to promote the development of local education and rural teachers in a comprehensive way, thereby preventing the intergenerational transmission of poverty.

This study focused on a part-time five-month-long PDP that aimed to offer academic training courses to rural teachers. This program included both content knowledge (pronunciation, language skills, literature, etc.) and pedagogical content knowledge (ELT methodology, national curriculum, etc.) to cater to rural teachers’ needs. Trainee teachers and the local educational authorities were advised to report local teachers’ learning needs and teaching difficulties prior to the training program. Then, the teacher educators integrated the teachers’ needs and the difficulties in designing the training modules and encouraged participant teachers to form collaborative learning groups. These primary school English teachers were assigned to prepare lessons together and discuss their lesson plans during the training. The group of teacher educators consisted of professors and foreign teachers with rich teacher training experiences from one of the top teacher training universities in China, outstanding faculty and research staff members from local education bureaus, and some excellent English teachers from key primary schools across China.

Considering the coronavirus outbreak and the rural teachers’ intensive working schedule, this training program was delivered online. The training portion of the study comprised two sessions; participant teachers attended lectures and workshops during their summer vacations (stage 1) and on weekends during the autumn semester^2^ (stage 2). Several QQ^3^ groups were set up by teaching assistants^4^ who organized regular after-training English language practice and shared various award-winning teaching videos throughout the training.

### Participants

This study employed purposeful sampling techniques with the aim of obtaining rich information that provides answers to the abovementioned research questions (Miles et al., 2014). At the outset of the study, an invitation for participation in this research was sent to all primary school English teachers who were participating in the five-month-long PDP. After they were given the information about the study, 52 teachers responded to our invitation and agreed to participate in this study. Among all the volunteer teachers, the researchers selected 46 from various poverty-stricken mountainous counties at the national level, including 34 female teachers and 12 male teachers. The participants were selected based on three criteria: (1) having various personal backgrounds and professional experience, (2) being recognized as information-rich individual cases who could provide collections of stories, and (3) having relatively low wellbeing at work.

Approximately 90% of the participant teachers already had an undergraduate degree (including an upgraded bachelor’s degree). However, their majors may not have been English or English education. Their teaching experience ranged from 0.5 to 35 years. These teachers’ classes are usually overcrowded, with at least 50 pupils who usually spend hours walking to school. Most of the participant teachers are required to regularly visit these students’ families in mountainous villages in order to persuade parents not to keep their children at home to work. Both authors worked as training educators for the participants.

### Research Method

It is suggested that wellbeing be measured subjectively, such as using self-report (Seligman, 2011). In the current study, to comprehensively investigate participant teachers’ wellbeing over time, the narrative inquiry approach was employed to explore teachers’ learning experiences and how their wellbeing was affected by such experiences during the program. Li and Craig (2019, p. 922) claimed that the narrative inquiry approach is the “first and foremost” way to study the inner world of teachers’ minds and to understand the nature of teachers’ thoughts about their professional lives in an underdeveloped social community. In most cases, only significant personal anecdotes could be recalled; therefore, by writing down their selective emphases, teachers could unite themselves closely with what they perceived (White, 2016; Yuan and Lee, 2016; Liu et al., 2019).

### Data Collection

The narrative frame, a written template consisting of incomplete sentences and blank spaces to guide participants’ writing of their stories, proved to be an appropriate tool to gather data in the narrative research (Benson, 2014). The current study provided teachers with a narrative frame to help them structure their stories in teacher diary entries in order to investigate their experiences in relation to their professional wellbeing. This approach helped to reduce teachers’ anxiety and they felt rather comfortable sharing potentially strong emotional reactions and experiences as it was uninfluenced by their peer colleagues (Acheson et al., 2016). The participants were required to record their rich and unique experiences at work and to voice their own feelings and thoughts about teaching practices, particularly stories related to their choice of a teaching career and their personal feelings about teaching rural students before and after participating in the current PDP. All their stories were written in Chinese, the native language of both the participants and the researchers.

Two rounds of diaries were collected through email and QQ groups by a research assistant. The first round of data collection was conducted immediately before the PDP commenced. The second round of data collection was conducted after the program finished. Ultimately, 98 teacher diary entries representing teachers’ inner thoughts about their professional wellbeing were collected (six teachers volunteered to write one more diary after completing stage 1), with a total of approximately 76,865 Chinese characters.

### Data Analysis

The two data collection phases yielded two large datasets that were manually analyzed and examined for emergent themes and patterns. Following the research questions and the abovementioned three-pillar model, a content analysis was conducted (Barkhuizen et al., 2014). A color-coding system was employed, when reviewing the scripts line by line. Expressions such as “I truly like being a teacher” and “Teaching is my dream career” were initially labeled as “interested in teaching,” while the expressions “I like English” and “English is very interesting” were labeled as “interested in the English language.” Then, these two codes were ultimately labeled as “interest-related” and categorized as meanings derived from work. All the codes that emerged were then developed and compared to form themes pertinent to the research questions; thus, possible patterns or themes became noticeable across the participants.

To establish reliability, all the participant teachers were informed that their data would be anonymously collected and analyzed. Participant teachers were advised to use a code name, such as WXJ, in their diaries, and all the teacher diaries were collected by the research assistant. Additionally, to avoid cultural politeness, the first researcher attempted to establish a rapport with the teachers both in and out of training and encouraged them to express their true feelings while writing their stories. In the meantime, to ensure the validity of this study’s interpretations, all the existing data were subject to multiple readings, particularly when the interpretation of data was uncertain (Gao and Xu, 2014). The researchers came together to refine the coding process and generate initial themes. Prior to settling on the themes, unclear issues that emerged in the preliminary interpretation of the data were sent back to the participants for further clarification. Moreover, to facilitate data interpretation, researcher triangulation was achieved through critical discussion among the two researchers and one research assistant regarding the teachers’ shared stories (Yuan and Lee, 2016).

## Results

As discussed above, participant teachers’ stories were analyzed regarding the impacts of the PDP and its contributory features that influenced teachers’ professional wellbeing. In general, changes in meanings that participant teachers derived from work, their professional engagement and their achievement were observed to various extents.

### Impacts of the Professional Development Program on Teachers’ Professional Wellbeing

#### Changes in Meanings That Derive From Being a Teacher

By viewing the participants’ stories that were collected before and after taking the current PDP, it was found that the meanings that rural teachers derived from being a teacher underwent a structural change, i.e., from being “interest and survival dominant” to the “interest-promotion-achievement triangulated mode.” Furthermore, two new types of meanings emerged, namely, responsibility meaning (i.e., the meaning relates to their sense of responsibility to local students) and instrumental meaning (i.e., the meaning relates to students’ ability to use English as a communication tool).

**FIGURE 1:**
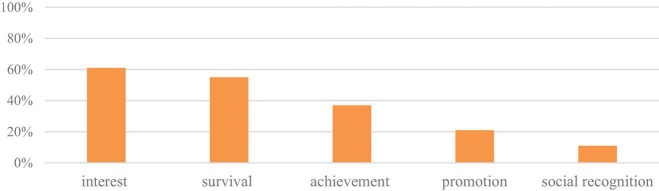
Meanings derived from teaching before taking PDP.

Prior to the training, teachers’ identified meanings from teaching were moderately simple, as shown in Figure 1.

Over 3/5 of teachers mentioned in their narratives that the major meaning they derive from being a teacher is to conform to their personal interests. For instance, teacher LL’s life dream was to become an English teacher like her own, and her school provided her with the opportunity to accomplish the dream.

##### Extract 1

I had a caring English teacher, and she influenced me a lot. My childhood dream was to become an English teacher like her. My school made my dream come true. (LL-1)

Nearly 50% of teachers consider teaching in rural schools to be a job that fulfills their survival needs, as it is “a stable job,” “close to home,” and “convenient for looking after the family.” Such jobs are more practical, such as teacher ZYY, who prefers to avoid any precarious situations.

##### Extract 2

I was born in this village. If I work here, I can stay with my family… Additionally, my job brings me good salary. I feel satisfied. I don’t like my life full of insecurity. (ZYY-1)

Approximately 37% of teachers serving in rural schools consider their students’ learning achievements to be the significant meaning of being a teacher. Teachers, such as WW1, feel strongly motivated when they feel they can help local students make progress.

##### Extract 3

Rural students’ English competence is relatively low compared with that of their urban counterparts. I’m very excited when I see their progress in exams. (WW1-1)

Additionally, 21% of participants emphasized their pursuit of promotion at work, such as “getting job promotion opportunities” (LY-1). Nearly 11% of teachers identified the importance of being socially recognized, such as “being a teacher here means being honored by the local community” (ZLQ-1) or “some illiterate parents brought their kids to school and showed great trust in me” (XX-1).

**FIGURE 2:**
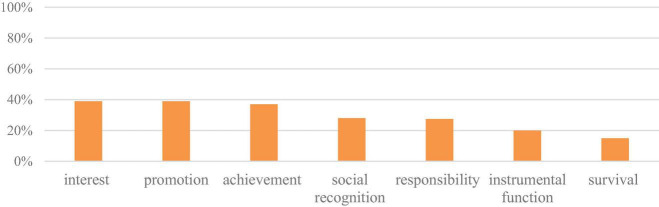
Meanings derived from teaching after taking PDP.

Remarkable changes in the meanings that teachers derive from their work could be identified after teachers attended the PDP. Their leading types of meaning became rather diverse, namely, interest, promotion and achievement, which were mentioned by up to 40% of participant rural English teachers; in addition, two new meaning types were discovered, namely, responsibility meaning and instrumental meaning, as seen in Figure 2.

In terms of responsibility meaning, as shown in Extract 4, teacher RX aims to bring her students a high-quality English learning experience. Similar to RX, another 11 participating rural teachers noted their responsibility to bring global visions to rural students.

##### Extract 4

In our QQ group, the teacher educator shared a video. When discussing with other peers the gains from this video, I started to reflect on the true value of my career. I see it as my responsibility to bring local students more beneficial language learning experiences. I now feel more gratified with my job. (RX-2)

In addition, in Extract 5, teacher LYY says that she considers the English language a tool that allows students to communicate with the world. This instrumental function was also addressed by nine other participants.

##### Extract 5

I wish my students could someday have the “pass,” to use English to know about the world and communicate with people from different countries… I feel passionate at work now. (LYY-2)

The teachers who identified the responsibility meaning and the instrumental meaning in their jobs showed positive emotions at work. Despite these newly emerged meanings, two more differences were found within the teachers’ narratives, namely, the survival meaning turned out to be less prevalent, while the social recognition meaning was almost trebled in teachers’ narratives, as shown in Extract 6.

##### Extract 6

I noticed the importance of fitting in to the local society. I received much positive feedback from local people. Parents are satisfied with my work. I am motivated to move on. (MD-2)

By taking the PDP, it seemed that some rural teachers, such as teacher MD, became eager to be approved by the local community. They tended to focus on their inherent and intrinsic selves instead of transient and unsustainable meanings in their jobs.

In general, after attending the training program, the meanings that teachers derived from their work experienced a structural change. The types of meaning became diversified, and new meanings emerged, which raised teachers’ positive emotional vibrations at work.

#### Changes in Engagement at Work

**FIGURE 3:**
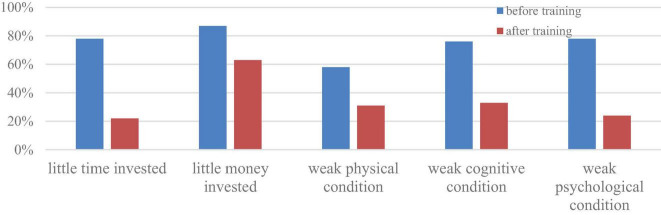
Rural teachers’ engagement at work before and after taking the PDP.

In this study, most teachers’ professional engagement was largely influenced by the PDP. Based on the analysis of rural teachers’ stories, two major indicators of the teachers’ engagement at work have been identified, namely, the resource commitment of invested time and money and the individual commitment of physical, cognitive and psychological conditions, as illustrated in Figure 3. In general, teachers’ professional engagement improved after attending the PDP.

Nearly 80% of the teachers emphasized their small time investment in their professional development, and more than 85% of them highlighted their small monetary investment in such development. As shown in Extract 7 and Extract 8, teacher SLL and teacher OY worked mechanically with limited time and money spent on improving their teaching practice. Their workload consequently consumed their time and attention.

##### Extract 7

I have little time to think of how to teach better. I’m very frustrated. I need to visit the students in extremely poor conditions and finish my poverty alleviation tasks after work. (SLL-1)

##### Extract 8

I don’t truly spend any money on professional development. I think the textbooks and reference books are totally enough for me. I feel drained every day with my overload of work. (OY-1)

After attending the training, approximately 80% of teachers claimed the amount of time they invested in self-development had increased, and nearly 40% of them claimed to spend money on upgrading themselves. As teacher CZR reported in Extract 9, she has invested time and money to pursue her academic development.

##### Extract 9

I felt very satisfied with my progress in spoken English. I bought some CDs to keep practicing my pronunciation during fragmented periods of time. I continue to learn new things. (CZR-2)

In addition, before attending the PDP, approximately 58% of teachers reported experiencing physical tiredness at work. In Extract 10, teacher SCH’s poor adjustment to her physical condition seriously affected her teaching and students.

##### Extract 10

My body condition was truly bad… It sometimes affected my students. I apologize for that. (SCH-1)

Similarly, approximately 80% of rural teachers claimed that their weak psychological conditions were due to various issues encountered at work. For instance, in Extract 11, teacher ZQ revealed her isolated anxiety about working with insufficient levels of support in teaching English. Such isolation explicitly drives teachers to accumulate passive emotions and gradually leads to mental fatigue and job lassitude.

##### Extract 11

Our school has 69 teachers, only two are English teachers…most of the time, there are no teams. I feel truly anxious and helpless. (ZQ-1)

In addition, similar to over 3/4 of the other teachers, teacher LL2 reported experiencing cognitive deficiency and low self-efficacy when he could not cope with the work standard in English teaching. It seemed that the destruction of agency at work turned these rural teachers into emotionally vulnerable individuals and thus had a negative impact on their wellbeing (O’Brien and Guiney, 2021).

##### Extract 12

My job is very demanding. I have to teach third-grade and fifth-grade in the same class. I used to be a Chinese teacher, so I’m always run-down and disappointed with my English ability. (LL2-1)

After attending the online training courses, a large difference could be identified in teachers’ stories. Most teachers, to a certain extent, expressed changes in the ways in which they work in rural schools.

##### Extract 13

I think the teacher educator’s living style is correct. It is important to keep fit, not only for working but also for myself. Now, I exercise after work every other day. It’s terrific. (ZQQ-2)

##### Extract 14

I truly enjoyed this training. It provides more content knowledge and teaching strategies that can be used in class. Although I have to face many problems at work, I’m not scared; I feel more proactive. (MTQ-2)

##### Extract 15

Although my work is still very challenging, I enjoyed the collaboration with peers and the support received from teaching assistants and teacher educators. They made me more passionate about teaching. (DXX-2)

As seen from the above extracts, the illustrations of weak physical, cognitive, and psychological conditions decreased in teachers’ diaries. For instance, teacher ZQQ took action to keep fit, while teacher MTQ addressed his progress related to his professional knowledge, which allowed him to embrace his vulnerability. In Extract 15, teacher DXX received various forms of support, which inspired her to devote herself to teaching practices, regardless of the demanding workload. In general, even though there was little change in the working conditions, almost all the participant teachers attempted to move forward in a vigorous manner and thus felt delighted and satisfied.

However, four teachers still articulated total passive engagement and adverse emotions. Teacher ZW2 was overwhelmed by the “redundant” work and felt that she had lost her real self. Additionally, similar to most of the new-to-career teachers, teacher LL1 had to prioritize her social and cultural clashes in rural environments and could rarely spend time on her self-development. Such an ambivalent situation ultimately leads to negative outcomes on teachers’ wellbeing at work.

##### Extract 16

I’m drowned by the schoolwork every day. The training sometimes clashes with my work. I can’t see what I’ll be like in 10 years. I’m helpless. (ZW2-2)

##### Extract 17

I need to get myself quickly involved in the local culture. As a new teacher, I had hoped I could have more time to develop my teaching skills, but no, never. I need to survive first. (LL1-2)

Moreover, two regressive changes were identified in the participants’ extra diaries. Among the six teachers who submitted one additional diary after completing stage 1 training, two of them who reported a positive change in professional engagement after summer training claimed to experience passive engagement at work by the end of the PDP.

##### Extract 18

In the new semester, all the work has pressed down on me with no time for breathing. I just want to lie down. (YW-3)

##### Extract 19

I tried to devote myself to teaching and learning, but finally failed. I was still in bad shape. Helpless. (SCH-3)

Teacher YW’s words revealed the fact that she was finally defeated by reality. Only during her vacation did she have time to enjoy professional development. Likewise, although teacher SCH once attempted to devote herself to teaching practice, her poor health finally discouraged her from working.

Overall, regardless of the abovementioned six exceptions, most of the participant teachers’ professional engagement grew in various ways. They recognized diversified values at work that could extricate them from underappreciated emotions and enhance their confidence in their teaching practice.

#### Changes in Achievement at Work

In terms of teachers’ professional achievement, there is a significant change identified in teachers’ scripts. Preceding the PDP, only two newly recruited teachers mentioned their professional development, and one stated his concern about job promotion. Almost all the participant teachers claimed that their sense of professional achievement mostly relied on students’ examination results.

##### Extract 20

When my students failed any test, I felt a strong sense of defeat. The dissatisfaction from the school authority and students’ parents made me depressed. (WW-1)

##### Extract 21

My students’ end-of-term exam results are displeasing. I’m very frustrated, and I don’t know how to help them to get better grades. (CX-1)

From the above extracts, it is seen that teachers’ wellbeing at work is highly related to students’ academic success. Both teacher WW and teacher CX reported their defeated emotions because of their students’ underachievement in examinations. Such claims are very common throughout the teachers’ narratives. For a long time, many people in rural areas have held the biased perception that only good grades on examinations can increase their children’s competitiveness in society and move them “upward in the social hierarchy” (Gao and Xu, 2014, p. 162). Thus, rural English teachers were easily marginalized if they failed to raise the students’ examination grades.

**FIGURE 4:**
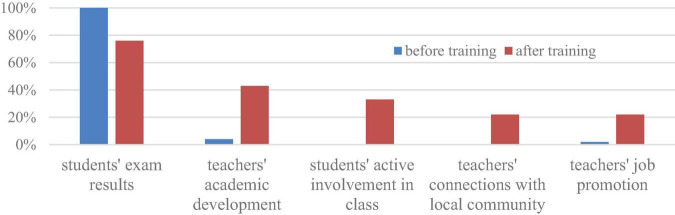
Rural teachers’ achievement at work before and after taking the PDP.

These attitudes shifted significantly after the PDP. Although approximately 76% of the participants still addressed their accomplishments regarding students’ exam results, new themes emerged. Over 33% of the participants felt a sense of achievement with their students’ active involvement in class, while 22% of them related the achievements to their connections with the local community. Moreover, the number of teachers who emphasized their achievements with their own academic development increased from 4% to 43%, and the teachers who emphasized their job promotion achievement also increased from 2% to 22% (see Figure 4).

##### Extract 22

We care too much about students’ grades… The students’ involvement in class is much more important… When the pedagogical skills I learned from the lesson study work well, I turn out to be more confident. Hopefully, I could design more effective tasks for my students. (CX-2)

##### Extract 23

Doing research is no longer impossible for me. My [research] paper won the second prize in a teaching contest. I’m proud of myself … Although my work is still intensive, I can better handle the daily stress that work throws at me and spend time on my professional development. (GZ-2)

##### Extract 24

I now notice how I should connect to myself and the people around me. I’m not an onlooker but rather a participant. This close relationship allowed me to bring new ideas to local groups. I feel a sense of achievement in doing that. (XQ-2)

From the above extracts, teacher CX enjoys her students’ positive responses to the pedagogical skills she acquired from the lesson study, while teacher GZ spends additional time pursuing academic growth. Both teachers have been inspired to further their academic capacity proactively. They are thus more poised when facing stress and anxiety at work. Additionally, teacher XQ has realized the importance of connecting to the local residents. Such close connections have provided her with opportunities to alter local people’s obsolete thoughts.

Overall, after attending the training program, most teachers view their professional achievements in diversified dimensions. Instead of simply focusing on students’ examination outcomes, their attention has shifted to considering how to activate students in class, develop their own professional competence, understand their own visions deeply, and connect closely with local people and community. These expanded achievements have enriched teachers’ sources of wellbeing in their occupations.

#### Reasons for the Changes

Features of the current PDP, which have impacts on teachers’ professional wellbeing, have also been identified from narrative stories. According to the trainees, the PDP’s content is the major triggering factor of teachers’ changes.

##### Extract 25

I used to be quite slack. However, now the pronunciation practice has motivated me a lot, and I enjoy practicing it in my spare time. (MLW-2)

##### Extract 26

I tried the dialogic teaching strategy in my class. It worked well among my students. I used it to design a model lesson, and it got an award in a teaching contest. I am quite excited. (LW-2)

From the above extracts, teacher MLW enjoyed pronunciation training and engaged herself to continue self-study, while teacher LW received positive feedback on the newly acquired strategy and even received an award due to its integration into lesson design. Eighty-nine percent of the participants declared the same positive impact received from the training content.

In addition, over 65% of the participants, such as teacher ZCP and teacher PP, mentioned the teacher educators’ vigorous impacts, including their abundant knowledge, extensive teaching and research experience, and elegant lifestyles.

##### Extract 27

The teacher educators helped me a lot with my scattered thoughts on teaching rural students. They analyzed the textbook using thriving ideas. I truly admire their knowledge and experience. (ZCP-2)

##### Extract 28

I should learn to accept my shortcomings. The teacher educators showed us how to ease the emotional pain and embrace the difficulties. This process works, and I’m truly relaxed. (PP-2)

Approximately 52% of the teachers emphasized the benefits of implementing various training approaches. In Extract 29, teacher MX addressed the positive needs-oriented approach employed in the current PDP, as well as the effectiveness of the embedded lesson study. Additionally, more than 41% of rural English teachers appreciated the training session arrangements. In Extract 30, teacher JA showed her preference for the two-stage training arrangement, which provided her with more opportunities to internalize and practice the acquired knowledge in authentic classes.

##### Extract 29

We were asked to share our teaching difficulties before and during the trainings. Teacher educators then analyzed the cases and provided us with effective solutions. The lesson study allowed me to reflect on my teaching approaches in a collaborative way. (MX-2)

##### Extract 30

The five-month-long training program fits me well. If the modules were squeezed into my summer vacation, I may have had no time to try it out and obtain feedback. (JA-2)

Overall, sufficient training content, experienced teacher educators, effective training approaches, and appropriate schedule arrangement of the training sessions are all significant features that impact participant teachers’ professional wellbeing.

## Discussion

### Toward a Tortuous but Beneficial Path of Professional Wellbeing

**FIGURE 5:**
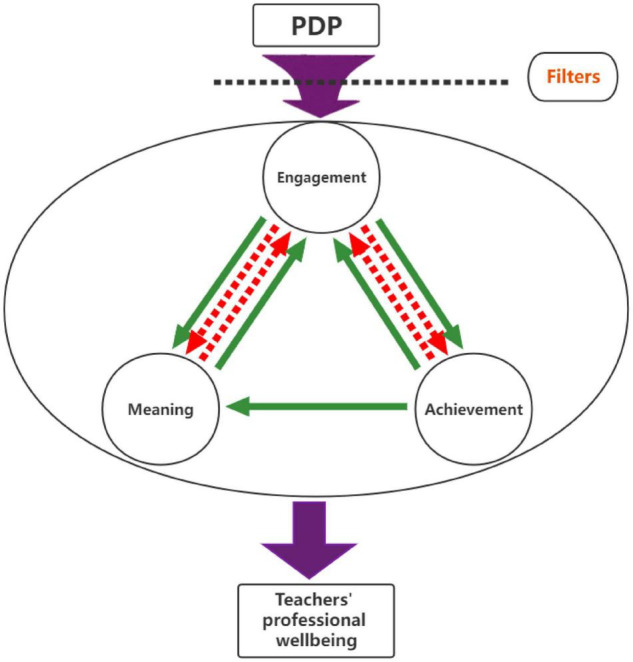
Indicators that triggered the change in teachers’ professional wellbeing.

Generally, the findings revealed that the current PDP functions well in altering rural primary school English teachers’ professional wellbeing in terms of the meanings they derive from teaching, their engagement and their sense of achievement. Figure 5 illustrates how each indicator is interrelated with one another and how they conjointly work to improve teachers’ professional wellbeing.

Above all, rural teachers’ achievement and engagement at work interplays with each other. After taking the PDP, rural teachers’ focus on their achievement shifted from students’ examination results to a variety of aspects. They attached great importance to their students’ gains in diverse ways and extended their perspectives on professional achievements to their own academic excellence, professional pursuits, and emotional connections, which therefore uplifted their students’ exam results in a natural manner. Such change strongly affected the participant teachers’ engagement at work. For example, along with the shift in which teachers related their professional achievements to their own academic growth, such as teacher GZ, they realized the significance of engaging in intellectual self-development. Therefore, although they still faced a complex educational context, these teachers attempted to allocate their physical, cognitive and emotional energies to upgrading themselves. Correspondingly, as the teachers strove to enhance their academic strength, they were more likely to reach a higher level of achievement at work, as shown in O’Brien and Guiney (2021). Gradually, these teachers were able to build up powerful strength in underprivileged conditions and to extricate themselves from emotional vulnerability.

In addition, teachers’ job engagement and the meanings they derived from work were tightly connected with each other. The diversified meanings that teachers drew from their jobs brought them stronger drives to serve in underdeveloped circumstances. Prior to the training program, the meanings possessed potential risks since the prominent interest and survival meanings were unstable by nature. In a challenging environment, where rural teachers tend to have a low level of ambiguous tolerance for professional practice, personal interest can easily vanish with poor conditions and mental exhaustion; teachers’ survival needs are also explicitly dynamic consistent with their fluctuating personal conditions, such as a change in marital status or other demographic transitions. However, the newly emerged responsibility meaning and instrumental meaning could easily bring about teachers’ positive appraisal with respect to their occupation. Even though these two meaning types are rarely mentioned in previous studies, from the perspective of the teachers’ narratives, these two meanings not only fulfill teachers’ commitment to work but also encourage them to fully engage in teaching and “make a difference” in a sustainable way. Similarly, teachers’ increased engagement at work helps them to discover various meanings from their teaching, including creating career progression opportunities and realizing personal aspirations. For instance, the current PDP established a collaborative learning environment for participant teachers. The more engaged these teachers were in the collaboration (e.g., teacher RX), the more meaning they drew from their work. Thus, these rural teachers were easily prevented from experiencing depression at work.

Furthermore, teachers’ achievement at work is also related to the meanings that teachers elicit from work. It is not a two-way relationship, as teachers may not achieve their goals by simply relying on the meanings they derive from work. Instead, through the PDP, teachers’ ultimate accomplishments could facilitate their acquisition of refreshing meanings from their everyday practice. For instance, by taking this PDP, some teachers, such as teacher XQ, successfully created a strong sense of community with local people. Such a growing awareness of rural membership develops a robust and enduring internal force that helps teachers notice how their profession is perceived in society. They are therefore inspired to proactively pursue stronger social recognition in local communities and go beyond “surviving” to truly “thriving” at work. In accordance with O’Brien and Guiney (2021), such teacher agency would then effectively alleviate rural teachers’ working stress and lift them out of unhappiness.

However, the impacts of the PDP on teachers’ professional wellbeing were not always positive in the current study. There were a few participant teachers who remained unchanged or even reversely changed after taking this program. One of the substantial causes of such an issue is that there are filters that directly interfere with the effectiveness of PDP. The action of these filters varies, but the main variation is related to engagement. Seen from the teachers’ narratives, their weak engagement and cognitive weariness hinders them from finding true meanings from their work and thus diminishes their ultimate professional achievement. Likewise, the counterforce from meaning and achievement also fails to have an effect on teachers’ engagement at work. It is found that the major filter that bars the action of the PDP is the insufficient level of support teachers receive from local communities. Working in underresourced ethnic minority regions, extra support, such as sufficient supportive access to resources, is necessary to accommodate teachers. Otherwise, these teachers may easily slip into low-morale situations and experience the dilemma of whether to continue their teaching careers in hinterland areas. Such ambivalence is “an indicator of lacking occupational wellbeing and can have deleterious effects for [teachers]” (Lauermann and König, 2016, p. 18).

In general, different from previous research such as Tang (2018) and Mercer (2020), the current study has revealed how the three indicators work on teachers’ changes in professional wellbeing. Although “twists and turns” exist, most rural primary school English teachers’ level of professional wellbeing was enhanced under the intertwined function of diversified meanings at work, growing job engagement and multivariate professional achievements after taking the two-stage PDP. Preceding the training, nearly all the participating English teachers expressed their emotional anxiety, physical exhaustion and even psychological burnout while working in rural areas. This situation was explicitly addressed and ameliorated, along with teachers’ evoked inner force gained through the PDP.

### A Demand-Initiated and Tailor-Made Professional Development Program Pattern

**FIGURE 6:**
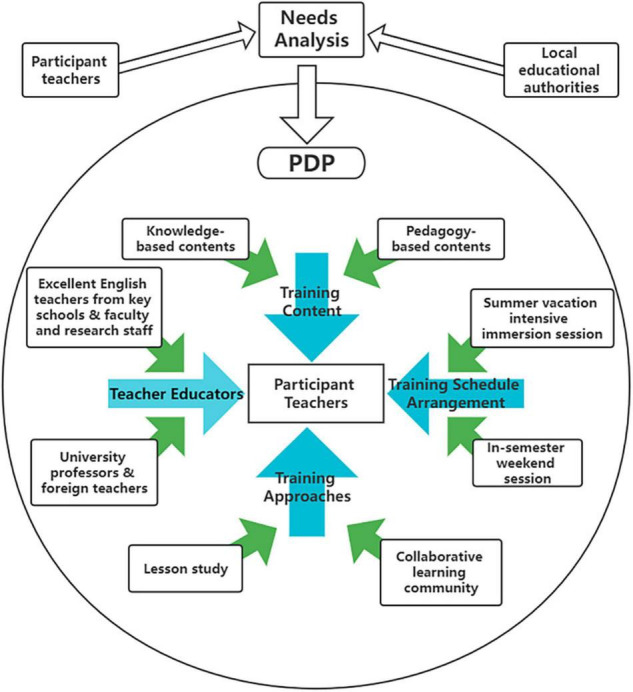
A demand-initiated and tailor-made PDP pattern.

When further investigating what leads to the beneficial changes in teachers’ professional wellbeing in underresourced rural settings, a demand-initiated and tailor-made PDP pattern emerged. Figure 6 explicitly illustrates all the emerged PDP features that affected teachers’ changes in professional wellbeing.

First, the training content and approaches are demand-initiated. As reported by Yan and He (2011), rural teachers’ typical learning needs are not always considered in the planning of professional development programs. Therefore, early communication on training needs was arranged with participant teachers and local educational authorities prior to the PDP. This approach largely avoided the problems that have been identified in previous training programs whose contents were either too broad or even irrelevant to many of the attending teachers (e.g., Li et al., 2020). Responding to rural teachers’ scarce subject knowledge and teaching strategies, both knowledge-based and pedagogy-based contents were prepared in the current training. Instead of emphasizing overly academic content, the current PDP prepared targeted resources that had high levels of relevance to rural teachers’ needs and spotlighted how theoretical knowledge could be integrated into rural classes. In terms of training approaches, the high demands on collaborative learning communities have been emphasized both in the literature (e.g., Mercer, 2020) and by participant teachers. Considering the isolated learning environment in remote schools, the current PDP creates a dynamic collaborative learning community to allow participant teachers to obtain support for their English language skills from teacher assistants, prompt and actionable feedback on unique classroom challenges from teacher educators, and positive emotional connections with peer colleagues. Developing “a network for professional learning communities” (Liu et al., 2021, p. 11) enables these rural teachers to learn from one another and to connect during times of celebration and turbulence. When coping with the increased complexity and more ambiguous accountability at work, this learning community could help mitigate teachers’ sense of anxiety and facilitate their working gratification (Mafora, 2013).

Additionally, the current PDP is tailor-made. The prepared resources used in the training content are typically selected for the participant teachers. For instance, in the module of teaching practice, authentic lesson plans were shared and discussed according to rural students’ characteristics. The training approaches were also custom fitted. A lesson study was conducted, which allowed teachers to collaboratively work on an in-depth exploration of an individual lesson designed for their students. This approach expanded participant teachers’ understanding of what learning is and how it works in rural contexts. In addition, the adopted flexible and multistage scheduling suited the participant teachers well. Since all of them work as in-service teachers in remote regions, arranging a one-shot summer vacation training could hardly solve their encountered practical problems in everyday teaching. Thus, the current PDP was scheduled as a summertime intensive immersion session with weekend sessions subsequently scheduled during the autumn semester. Such a two-stage arrangement allowed teachers to understand, practice, reflect on and internalize the acquired knowledge and then transform their inappropriate teaching beliefs into more appropriate beliefs. As the PDP spanned five months, these rural teachers could always review their classroom teaching habits and bring newly occurring issues to discuss in the training. Such consistent help for rural teachers indeed led to enhanced job satisfaction.

Likewise, the team of teacher educators was also tailor-made for the participating rural teachers. Some teacher educators, who were senior teachers from key schools located across China, shared stories about their successful teaching experience stories in the training, while other teacher educators, who were top university professors with abundant knowledge and experience, not only delivered insightful ideas to the trainee teachers but also led to their increased awareness of the significance of self-development. Experienced foreign teachers were also involved in providing high-quality lessons in English language skills and cultural knowledge. More importantly, noticing the fact that teaching in isolated remote areas occasionally demotivates teachers’ passions at work and disengages them from their commitment, during the PDP, most teacher educators attempted to share their positive lifestyles and to enlighten rural teachers to pursue the best version of themselves. Regarding teacher educators as their role models, these participant teachers strove to change, particularly when they believed that what they were doing was of great consequence. To a large extent, the teacher educators helped awaken the rural teachers’ vigor, which had been destroyed by the clash between their personal and work lives in underdeveloped areas, and reactivated their social and emotional competencies. The participant teachers then easily felt empowered and emboldened in regard to their teaching.

Generally, a demand-initiated and tailor-made PDP is a catalyst that leads to rural teachers’ enhanced visions and beliefs toward the teaching profession. This shift fits the dynamic, changing phenomenon of rural schools, thereby resulting in teachers’ higher teaching ambitions and enabling them to be more sensitive to happiness (Tang, 2018). It is therefore beneficial to their professional wellbeing.

## Conclusion and Implication

According to the current study, rural English teachers’ professional wellbeing has been enhanced following the positive change of their derived meanings from being a teacher, their engagement and their sense of achievement at work. In most cases, this change occurred due to the demand-initiated and tailor-made PDP. The issues we have explored herein should provide insights to people who are concerned with similar issues in other educational and cultural settings; this study provides insight into teacher retention in deep-poverty schools located in high-needs areas facing similar challenges by tracking teachers’ changes in wellbeing in transformative ways (White, 2016).

With regard to certain teachers’ negative engagement that has been identified in the current PDP, it is suggested that PDP organizers should work more closely with local educational authorities and rural schools to provide consistent support in managing the challenges encountered during PDPs (Gregersen et al., 2020; Mercer and Gregersen, 2020; Cece et al., 2022). For instance, teachers from such remote schools could take turns attending the training program, and the participant teachers’ additional work could be allocated to other colleagues; in return, the teachers who received the training opportunities should share their acquired knowledge through regular teambuilding exercises. Additionally, when arranging an online training program, the issue of how to stimulate and monitor participants’ engagement in a beneficial manner should be discussed further.

In conclusion, education plays an essential role in poverty alleviation and rural vitalization, particularly in hinterland areas where educational resources are understandably limited. Teachers are the key force to guarantee that change happens in these areas. Although the current PDP helped a majority of rural primary school English teachers improve their professional competencies and regain true happiness in regard to teaching, it would be more efficacious to foster a positive, psychology-based training program that could enhance rural teachers’ sense of wellbeing in these remote regions. More studies using different kinds of research instruments are encouraged to explore the multiple complexities of teacher experience and teachers’ actual wellbeing.

## Data Availability Statement

The original contributions presented in this study are included in the article/supplementary material, further inquiries can be directed to the corresponding author/s.

## Ethics Statement

Ethical review and approval was not required for the study on human participants in accordance with the local legislation and institutional requirements. The participants provided their written informed consent to participate in this study.

## Author Contributions

XW: whole research design and data collection and analysis. ZC: program arrangement and data analysis. Both authors contributed to the article and approved the submitted version.

## Conflict of Interest

The authors declare that the research was conducted in the absence of any commercial or financial relationships that could be construed as a potential conflict of interest.

## Publisher’s Note

All claims expressed in this article are solely those of the authors and do not necessarily represent those of their affiliated organizations, or those of the publisher, the editors and the reviewers. Any product that may be evaluated in this article, or claim that may be made by its manufacturer, is not guaranteed or endorsed by the publisher.

## Footnotes

1 The deep poverty areas in China are announced by the Leading Group Office of Poverty Alleviation and Development (of the State Council).

2 These teachers worked full time at schools in the autumn semester.

3 QQ is a free communication and social-messaging application.

4 The teaching assistants are postgraduate students from one of the top universities in China, who majored in English language teaching.
